# The Gastrointestinal Microbiome and Musculoskeletal Diseases: A Beneficial Role for Probiotics and Prebiotics

**DOI:** 10.3390/pathogens2040606

**Published:** 2013-11-14

**Authors:** Luis Vitetta, Samantha Coulson, Anthony W. Linnane, Henry Butt

**Affiliations:** 1 Medlab, Sydney 2015, Australia; E-Mail: tlinnane.cmbm@live.com.au; 2 School of Medicine, The University of Queensland, Brisbane 4102, Australia; E-Mail: s.coulson@uq.edu.au; 3 Monash University, Melbourne 3800, Australia; 4 Bioscreen, Bio21, The University of Melbourne, Melbourne 3010, Australia; E-Mail: hbutt@unimelb.edu.au

**Keywords:** gastrointestinal tract, osteoarthritis, microbiome, prebiotics, probiotics, inflammation

## Abstract

Natural medicines are an attractive option for patients diagnosed with common and debilitating musculoskeletal diseases such as Osteoarthritis (OA) or Rheumatoid Arthritis (RA). The high rate of self-medication with natural products is due to (1) lack of an available cure and (2) serious adverse events associated with chronic use of pharmaceutical medications in particular non-steroidal anti-inflammatory drugs (NSAIDs) and high dose paracetamol. Pharmaceuticals to treat pain may disrupt gastrointestinal (GIT) barrier integrity inducing GIT inflammation and a state of and hyper-permeability. Probiotics and prebiotics may comprise plausible therapeutic options that can restore GIT barrier functionality and down regulate pro-inflammatory mediators by modulating the activity of, for example, *Clostridia* species known to induce pro-inflammatory mediators. The effect may comprise the rescue of gut barrier physiological function. A postulated requirement has been the abrogation of free radical formation by numerous natural antioxidant molecules in order to improve musculoskeletal health outcomes, this notion in our view, is in error. The production of reactive oxygen species (ROS) in different anatomical environments including the GIT by the epithelial lining and the commensal microbe cohort is a regulated process, leading to the formation of hydrogen peroxide which is now well recognized as an essential second messenger required for normal cellular homeostasis and physiological function. The GIT commensal profile that tolerates the host does so by regulating pro-inflammatory and anti-inflammatory GIT mucosal actions through the activity of ROS signaling thereby controlling the activity of pathogenic bacterial species.

## 1. Introduction

Patients diagnosed with musculoskeletal diseases are reported to have a predisposition to GIT disturbances that includes dyspepsia, nausea, abdominal bloating and irregular bowel habits [1,2]. The intake of high-dose paracetamol (>2 g per day) and/or NSAIDs for musculoskeletal pain [3,4] has adverse effects on GIT physiology and morphology further inducing GIT symptoms reported by these patients [2,5,6,7,8]. GIT commensal microbial viability and growth may potentially be disrupted with the use of these medications, as demonstrated in animal and *in vitro* studies [9,10,11]. The association between GIT dysfunction, altered microbial profiles and the inconsistent clinical results for compounds such as glucosamine and green-lipped mussel extract in treating symptoms of OA has only recently been alluded to [12,13]. Biological compounds are a potential substrate for bacterial metabolism and transform before being absorbed across the GIT mucosa, potentially modifying the therapeutic activity that has been demonstrated *in vitro* [14,15,16,17,18]. Moreover, gut dysbiosis has also been correlated with rheumatoid arthritis [19]. GIT dysbiosis is a term used to describe bacterial imbalances usually in the GIT. Such imbalances may increase the risk of developing GIT barrier dysfunction, via enterocyte hyper-permeability [*leaky gut*] to bacterial endotoxins.

In this review/commentary, we advance the hypothesis that a dysbiotic GIT and the pathogenic commensal microbiome cohort play a significant role in the induction of pro-inflammatory mediators in musculoskeletal diseases. The disruption of the GIT epithelial barrier that can accompany chronic use of analgesic medications can exacerbate local inflammatory responses induced by bacterial species that further disrupt GIT physiological function leading to unregulated inflammatory responses.

## 2. The Gastrointestinal Tract (GIT) Microbiome

The GIT microbiota comprise one of the most metabolically and immunologically active organs, outnumbering human cells 10-fold and encoding 100-fold more unique genes than that of the human genome [20,21,22]. The GIT is the site at which microbiota and environmental antigens are most exposed to the immune system. The symbiotic relationship between GIT microbiota and the host’s immune system begin at birth, with the microbiota shaping immune system development, and conversely the immune system shaping microbiota composition [23]. In the absence of microbes, neither the host gut physiology nor the immune system develops normally [24]. GIT microbiota are highly structured and exert extensive protective, structural, metabolic and immune influences within and systemically from the gut [15,17]. Microbiota communicate with the host’s immune system and beyond through signaling pathways interacting with organs such as the gut, liver, muscle and brain, together comprising a series of host-microbe metabolic axes [23]. This allows interactive, multi-directional chemical communication between organs and a series of microbial species that can modulate metabolic reactions. 

Among the 100 different bacterial phyla so far identified on our planet, only five to six are located within the human GIT. *Bacteroidetes*, *Firmicutes*, *Actinobacteria*, *Proteobacteria*, Fusobacteria and *Verrucomicrobia* represent the dominant phyla while less prevalent bacteria belong to the *Cyanobacteria*, *Fusobacteria*, *Lentisphaerae*, *Spirocjaetes* and TM7 phyla [25,26]. Microbial colonisation ranges from 10^2^ CFU/g in the proximal gut, increasing in number and diversity to 10^12^ CFU/g in the distal gut [26]. The abundance of each dominant phyla remains consistent within adults; however, up to 70% of bacterial subsets within the phyla groups are specific to each individual [26]. Furthermore, the phylogenetic and functional composition of the individual microbiota do not remain stable, but instead demonstrate varying degrees of plasticity in response to diet, environment and geographical location. Recent studies report that GIT microbial flora predominance consists of three dominant enterotypes characterized as *Bacteroides*, *Prevotella*, and *Ruminococcus* species [27,28]. Moreover that the GIT microbiome is subject to modification by dietary and environmental variables as shown by altered patterns of enterotype dominance [28]. They further reported that faecal communities are clustered into the enterotypes *Bacteroides* and *Prevotella*. Enterotypes were strongly associated with long-term diets, particularly high protein and animal fat (*Bacteroides*) *versus* low protein and higher carbohydrates (*Prevotella*). A controlled-feeding study (from 10 subjects) showed that microbiome composition changed detectably within 24 h of initiating a high-fat/low-fiber or low-fat/high-fiber diet, but that enterotype identity remained stable during the 10-day clinical study, indicating that alternative enterotype states are associated with long-term dietary patterns only.

## 3. The Gastrointestinal Tract (GIT) Microbiome and Immune Regulation

The intestinal microbiota can have marked effects on mucosal defense mechanisms (*i.e.*, competition for mucosal colonisation and metabolic substrates plus synthesis of regulatory factors such as short chain fatty acids and bacteriocins) and the innate and adaptive immune responses of the host, and are therefore integral to maintaining immune homeostasis within the developing and adult gut [29,30]. Commensal bacteria, however, differ in their ability both to promote development of the gut-associated lymphoid tissues and to maintain its function [29,30].

The mammalian GIT microbiota extensively interacts with the host via the intestinal mucosal surface, a site with a complex and interactional environment that is continuously exposed to a range of commensal microorganisms. The GIT is often subjected to intrinsic and extrinsic insults that can significantly disrupt GIT homeostasis. As a consequence of the high metabolic load that is encountered in the GIT, toxins may arise due to adverse bacterial activity [31], or by dietary and environmental influences [32], and these can exact a heavy toll on the enterocyte’s barrier function. Dysbiosis is a GIT perturbation that can be induced by the administration of antibiotics, imprudent dietary practices, medications, immune deficits, inflammation and pathogenic infections leading to increased numbers of Gram-negative bacteria and translocation of their gene products such as lipopolysaccharides (LPS) across the inflamed, permeable intestinal epithelia generating systemic inflammation and endotoxaemia [33,34]. The GIT enterocyte in conjunction with saccharolytic bacteria (that is, those that predominantly ferment carbohydrates) or proteolytic bacteria (that is, those that predominantly utilize protein) [35], is exposed to an array of potentially toxic molecules that predispose to disease. 

The past six decades has seen a significant increase in the prevalence of autoimmune diseases [35,36] such as RA. This was the catalyst that led to the formulation of the hygiene hypothesis. Over the past two decades, the hygiene theory has been tested and tweaked, expanded and extended [36]. This hypothesis provides a biologically plausible explanation for the trend that implicates diminished exposure in early childhood to those *commensal* infections that boost immune defenses. This deficit subsequently enhances the risk, for later life, of GIT inflammatory problems that disrupt normal/regulated GIT inflammatory responses and increases the susceptibility to developing autoimmune diseases [37]. The hypothesis proposed a reduced exposure to infections in early childhood owing to a combination of diminishing family size and better personal hygiene, which might then go on to increase the risk of developing allergic diseases [38]. The interface of the microbial environment with the innate immune system could be significantly modulated so that its ability to impart instructions to adaptive/regulatory immune/inflammatory responses would be adversely affected, particularly when such interactions occurred *in utero* and or were presaged in early life. Researchers [37,38] documented this trend highlighting that an epidemic of both GIT autoimmune diseases in which the immune response was dominated by Th1 cells (such as Type 1 Diabetes Mellitus, Crohn’s disease, Multiple Sclerosis) or allergic diseases in which the immune response was dominated by Th2 cells (such as Asthma, Allergic Rhinitis, and Atopic Dermatitis) were becoming increasingly prevalent in Western communities. 

Evolution has naturally endowed the human species with immune/inflammatory regulatory mechanisms activated by the interactions with both the external and internal microbial environments. These then serve to fine-tune both Th1 and Th2 antigen-driven effector responses [38]. The innate immune system senses the environment and accordingly modulates the T regulatory arm, the ultimate keeper of the balance between antigen tolerance and responsiveness. The efficiency of the regulatory interface in its current state would paradoxically be jeopardized by a decrease in the microbial burden that the immune system has co-evolved with [38]. It is therefore a coordinated effort between host tolerance and bacterial immune regulation. Active sampling and immune responses to resident bacteria, pathogens and other antigens are mediated by enterocytes, microfold (M) cells and dendritic cells within the mucosal epithelium [15]. These immunosensory cells secrete chemokines, cytokines, anti-microbial peptides and secretory immunoglobulin A (sIgA) in response to enteric microbiota and pathogens [39]. Intestinal dendritic cells directly sample lumenal antigens [40] and present them to CD4^+^ T-helper cells, thereby regulating immune effectors such as antigen-specific CD8^+^ cytotoxic T cells and B cells, as well as non-antigen specific macrophages and eosinophils [41,42]. The enterocytes and dendritic cells express two major host pattern recognition receptor (PRR) proteins that are crucial for communicating immune cell activation in response to specific microbial antigens. They include the family of Toll-like receptors (TLRs) and nucleotide-binding oligomerization domain (NOD1 and NOD2) proteins that allow the host to differentiate the commensal from the pathogenic bacteria [15,43]. 

The range of receptor proteins bind to and become activated by various bacterial ligands including LPS, peptidoglycans, lipotechnoic acids, lipopeptides, flagellin and zymosan (from *yeast* cell walls) [15,28]. Upon activation, these receptor proteins trigger intracellular inflammatory signaling pathways. Dendritic cells modulate immune responsiveness or tolerance to the bacterial antigens by promoting either effector (Th1 or Th2) or regulatory T (Treg) cells. While most enteric bacteria predominantly reside in the mucus layer, *Clostridia*-related, Gram-positive bacteria known as Segmented Filamentous Bacteria (SFB), is able to anchor onto epithelial cells adjacent to M cells [44,45] and are the most potent modulators of colonic Treg cells [46]. Commensal-derived metabolites and the composition of bacterial surface structures have various impacts on the host immune system. Host inflammatory responses to pathogenic bacteria are centrally controlled by NF-κB, which is responsible for expression of chemokines and cytokines. Most commensal bacteria do not activate NF-κB, but rather certain species can restrain inflammatory signals in response pathogenic bacteria by limiting NF-κB activation [28,47]. Immunotolerance to commensal bacteria as seen in a healthy gut is in part due to the absence of the expression or the redistribution of TLRs from epithelial apical membranes along the length of the GIT, which is thought to limit the activation of TLRs by commensal bacteria and bacterial products [35]. Further, intestinal enterocytes express high levels of the TLR inhibitor Toll-interacting protein (Tollip) that is expressed constitutively or induced which limit TLR-mediated inflammatory responses. Tollip expression correlates directly with the luminal bacterial load *in vivo* and is highest in healthy colonic mucosa [15,47].

Studies exploring the molecular mechanisms that might underpin the hygiene hypothesis have focused mostly on the interactions between bacterial products and Toll-like receptors (TLRs)—the main transducers of microbial signals to the innate immune system and critical regulators of CD4 T-cell activation and regulation [48,49]. Therapeutically, a recent review has highlighted how in those individuals with chronic helminth infections there is often an association with a reduced prevalence of inflammatory disorders, including allergic diseases [50]. Mechanistically it was reported that by inducing or expanding regulatory B cells with helminths that this may then open novel avenues for the treatment of inflammatory diseases, such as allergic asthma [51].

## 4. The GIT Microbiome and Musculoskeletal Diseases

The implication of oral and intestinal microbiota in the development of RA was conceived more than a century ago, with more recent analytical techniques confirming altered intestinal microbial profiles in RA patients, providing a plausible aetiopathogenic role [52]. A recent study investigating the colonic microbial biocenosis and colonizing ability of opportunistic bacteria in 32 patients with RA and 30 healthy subjects reported RA was associated with significant modification of the GIT microbiota [19]. There were notable decreases in Lactobacteria and significant increases in *Enterococci*, *Clostridia*, *Colibacteria* genus with reduced enzymatic activity and an increase in opportunistic species when compared to the control subjects. Symbiotic interactions between species, was also altered, with *Bifidobacteria*, *Bacteroids*, and lacto-positive *Colibacteria* species reduced while the abundance of opportunistic *Enterobacteria* and *Staphylococci* were increased. Opportunistic *Enterobacteriaceae* were present in urine and nasal mucosa suggesting their translocation from the intestines. Consistent with these observations, it was concluded that changes in intestinal microflora and colonization by opportunistic bacteria enhance the risk of RA patients developing co-morbid conditions [19]. An earlier study, by Vaahtovuo *et al*. 2008 [53] supports the hypothesis the intestinal microbes participate in the aetiopathogenesis of RA. When comparing fecal microbial profiles between patients with early RA or fibromyalgia (FM) they found that RA patients had significantly less *Bifidobacteria* and bacteria of the *Bacteroides-Porphyromonas-Prevotella* group, *Bacteroides fragilis* subgroup, and *Eubacterium rectal-Clostridium coccoides* group compared to patients with FM as it is a non-inflammatory condition [53]. The hypothesis that GIT microbiota and their degradation products induce synovial inflammation and the development of arthritis in genetically susceptible individuals has previously been proposed by Toivanen [54] who states that constant seeding of bacterial products from the gut leads to synovial inflammation and chronic arthritis in susceptible people. 

Profiling of gut bacteria in patients diagnosed with OA is limited. Altered GIT bacterial profiles and gut functionality in patients diagnosed with OA has been reported by our group [12,13]. GIT symptoms such as reflux, nausea, abdominal bloating and pain, flatulence, constipation and diarrhea were reported by 18%–39% of study participants ranging from mild to very severe symptom severity. Fecal analysis demonstrated elevated counts of species from the genus *Enterococcus*, *Streptococcus*, *Staphylococcus*, *Eubacterium*, *Lactobacillus*, *Bifidobacterium* and *Clostridium*. Species from the genus Prevotella were only detected in 5% of the patients. The most prevalent species detected within each genera were: Coliforms (*Escherichia coli*, *Klebsiella pneumoniae*); *Enterococcus* (*E. faecalis*, *E. faecium*); *Streptococcus* (*S. mutans*, *S. parasanguinis*, *S. salivarius*); *Staphylococcus* (*S. aureus*, *S. epidermidis*, *S. capitis*); Yeasts (*Candida albicans*); *Bacteroides* (*B. vulgates*, *B. ovatus*, *B. fragilis*, *B. uniformis*, *B. thetaiotaomicron*, *B. stercoris*); *Eubacterium* (*Collinsella aerofaciens*); *Lactobacillus* (*L. acidophilus*, *L. gasseri*, *L. paracasei*, *L. fermentum*); *Bifidobacterium* (*B. longum*, *B. animalis*, *B. adolescentis*, *B. bifidum*); *Clostridium* (*C. innocuum*, *C. tertium*) with *C. perfringens* detected in one patient. After 12 weeks supplementation with either whole green-lipped mussel (GLM) extract or glucosamine sulfate, both groups demonstrated notable trends in a decrease of the genus *Clostridia* and *Staphylococcus*. This occurred concurrently with significant improvements in OA symptoms, namely joint pain, stiffness and function and furthermore, significant improvements in GIT symptoms such as bloating, abdominal pain, reflux and altered bowel habits. SFB are one of the most potent modulators of colonic regulatory T (Treg) cells and play an important role in the regulation of IL-17 synthesis from Th17 cells [55]. While microbiota-induced Th17 cytokines are crucial for protection against intestinal pathogens, they can also contribute to inflammation [56]. For example, SFB can initiate autoimmune arthritis in murine models via the induction and differentiation of antigen-specific Th17 cells with subsequent IL-17 production and auto-antibodies [55] and also induce intestinal inflammation [57].

Further to the involvement of bacteria and bacterial toxin systemic dissemination from the GIT to distal inflammatory sites within the body, the diverse colonization (>700 species identified) of bacteria in the oral cavity also serves as a possible source of inflammatory triggers thought to be involved in the aetiology of RA [58,59,60]. It is reported that patients with RA have a higher prevalence of periodontal disease (PD) compared to healthy controls, with the severity of periodontitis correlated to the severity of RA disease activity [60]. PD is caused by oral microbiota, in particular by certain a group of anaerobic Gram-positive bacteria, namely *Porphyromonas gingivalis*, *Treponema denticola*, and *Tannerella forsythia* that colonize the subgingival spaces as biofilms [61]. Oral bacterial DNA has been identified in the synovial fluid (SF) from RA patients’ in particular *P. gingivalas* and *Prevotella intermedia* [62]. Furthermore, high levels of antigen-specific antibodies against periodontal pathogens in RA and OA-SF namely against *Porphyromonas gingivalis*, *Prevotella intermedia and Bacteroides forsythus,* have also been reported [63]. The discovery of bacteria and bacteria-derived peptidoglycan-polysaccharides (PG-PS) in SF from not only reactive arthritis, but chronic forms of arthritis such as RA and OA, indicates that arthritic joints are not sterile as previously thought [64]. The presence of bacterial antigens within the synovial fluid triggers and exacerbates joint inflammation [64,65,66,67,68,69]. Other bacteria antigens detected in SF include *Pseudomonas* sp., *Shigella* sp., *Escherichia coli*, [67] *Streptococcus thermophilus*, *Chlamydia trachomatis* and *Chlamydia pneumonia* [68,69,70]. The dermis-derived bacteria, *Propionibacterium acne*, a gram-positive anaerobic bacillus, has recently been identified in shoulder joint fluid and tissue specimens from patients with radiographic signs of shoulder arthropathy (OA and cuff tear arthropathy) and also from patients with oligoarthritis, with a number of case studies supporting these findings [71,72] It has been reported that intra-articular injection of *P acnes* in rats leads to an erosive arthritis [73].

Environmental factors such as the composition and metabolic activity of the gut flora, immune system reactivity and genetic factors are all believed to play a role in the progression of gut inflammatory states for conditions such as RA [52]. Clinical observations suggest that certain intestinal and extra-intestinal bacterial infections may perhaps precede or reactivate chronic intestinal inflammation. A number of microbial agents have been implicated as initiating factors in the pathogenesis of for example inflammatory bowel disease (IBD), including *Mycobacterium paratuberculosis*, measles virus, *Listeria monocytogenes*, and adherent *E. coli* [74]. The plausible mechanism for causality is the disruption of the GIT microbiome. A proposed mechanism by which pathogenic micro-organisms may drive intestinal inflammation in susceptible individuals is via disruption of the mucosal barrier. This could then lead to an increased uptake of luminal antigens or mimics of self antigens and activate the mucosal immune system via modulation of transcription factors such as NFkB [74] by sustaining a dysregulated pro-inflammatory action. 

## 5. Probiotics and Prebiotics and Musculoskeletal Disease

### 5.1. Probiotics

Probiotics are living organisms in food and dietary supplements that upon ingestion can improve the health of the host beyond their inherent basic nutritional content [75]. A recent review has highlighted numerous clinical studies that have demonstrated efficacy in the treatment of inflammatory and immune deficit diseases and conditions [76].

The therapeutic potential of using probiotics to treat arthritic conditions has only recently been recognized, with a small number of animal and human studies having been undertaken, predominantly for autoimmune arthritic diseases. Beneficial effects have been demonstrated by probiotics controlling inflammatory diseases, although this is dependent on the species and strain of bacteria [77]. 

Oral administration of *Lactobacillus casei* (5 × 10^9^ CFU × 3 times per week) was shown to protect against RA progression in a rat model, whereby *L. casei* suppressed collagen-induced arthritis (CIA) and reduced paw swelling, lymphocyte infiltration and destruction of cartilage tissue [78]. *L. casei* reduced the pro-inflammatory cytokines (IL-1β, IL-2, IL-6, IL-12, IL-17, IFN-δ, TNF-α and COX-2) while up-regulating immunoregulatory IL-10 levels. The authors concluded that *L. casei* suppresses Th-1 immune responses of arthritic inflammation. This is supported by a further study demonstrating oral administration of *L. casei* (2 × 10^10^ CFU, 500 mg/kg body weight × 3 times per week) in combination with type II collagen (CII), synergistically suppressing arthritic inflammation (RA model in rats) by promoting oral tolerance, demonstrated by a decrease in pro-inflammatory cytokines (IL-1β, IL-2, IL-6, IL-12, IL-17, IFN-δ and TNF-α) and an increase in anti-inflammatory cytokines (Il-10 and TGF-β) and Foxp3^+^ CD4^+^ T cells which are one of the most important subset of regulatory cells [78]. CII is a major self-antigen that plays a key role in the T cell mediated immune responses of RA. More recently, *L. casei* (2 × 10^8^ CFU/mL of distilled water given daily) [79] demonstrated a protective effect against chronic inflammation and arthritis in a collagen-induced rat arthritis model preventing synovial infiltration, pannus formation, cartilage and bone destruction with a significant reduction in pro-inflammatory cytokines [79,80].

OA animal models have also been investigated with the administration of probiotics. In a rat OA model, the authors demonstrated that the oral administration of *L. casei* (2 × 10^10^ CFU/kg, 500 mg/kg of body weight) together with CII (250 mg/kg body weight) and glucosamine (250 mg/kg body weight) 6 times per week, more effectively reduced pain, cartilage destruction and lymphocyte infiltration that the treatment of GlcN or *L. casei* alone [81]. Co-administration of *L. casei* with GlcN significantly decreased nuclear translocation of NF-κB in chondrocytes, with a decrease in pro-inflammatory cytokines (IL-1β, IL-2, IL-6, IL-12, IL-17, TNF-α and IFN-δ) and MMP1, MMP3 and MMP13 and up-regulation of ant-inflammatory cytokines (IL-10 and IL-4). Glucosamine administered alone did not effectively suppress OA progression, but it still demonstrated anti-inflammatory activity as detected by cytokine analyses.

There are a small number of human clinical trials (Table 1) that have assessed the therapeutic efficacy of administering probiotics to patients with autoimmune arthritic diseases. However, there are no clinical studies that have investigated the role of probiotics in reducing the symptoms of OA. A recent animal study though has provided plausible data that a probiotic strain investigated, namely, *Lactobacillus casei* could act as a potent nutraceutical modulator for the treatment of OA. Pain was reduced, as were inflammatory responses, and articular cartilage degradation [81]. 

A small randomized, double blind, placebo controlled pilot study assessed the efficacy of *Lactobacillus rhamnosus GG* (5 × 10^9^ CFU/capsule) twice daily in 21 RA patients for 12 months duration [82]. There were no statistical differences in the clinical parameters, biochemical variables (e.g., CRP, ESR, pro- and anti-inflammatory cytokines) or Health Assessment Questionnaire (HAQ) index between the two groups over the study period. The probiotic group did report overall increased subjective wellbeing with a decrease in tender and swollen joints and decrease in disease activity (reduced by 71%) compared to the placebo group (reduced by 30%). In a double-blind, randomized placebo controlled study, 63 patients with spondyloarthritis (SpA) were treated with a probiotic containing *Streptoccocus salivarius* (1 × 10^8^ CFU/g), *Bifidobacterium lactis* (4 × 10^8^ CFU/g) and *Lactobacillus acidophilus* (1 × 10^8^ CFU/g) or a placebo at a dose of 0.8 g (1 level tea spoonful) twice daily for 12 weeks [83]. Probiotic therapy did not improve disease activity, function or quality of life in-patient with SpA but the results may have been due to trial duration, the species and strains of bacteria chosen as bacterial activity cannot be generalized. The probiotic group did however demonstrate an improvement in GIT symptoms within the 12-week period. A double-blind, randomized placebo controlled study assessed the efficacy of a probiotic containing *Lactobacillus rhamnosus* and *Lactobacillus reuteri* in treating 30 patients with RA for three months duration [84]. Each probiotic capsule contained 2 billion CFU with one capsule taken orally twice daily. Administration of probiotics did not demonstrate an improvement in disease activity compared to placebo, and while not significant there was a trend for the probiotic group to demonstrate reduced secretion of several pro-inflammatory cytokines (Il-1α, IL-6, IL-15 and TNF-α). Furthermore, in a double-blind, randomized placebo controlled study, 45 RA patients were supplemented with a probiotic containing *Bacillus coagulans* (2 billion CFU/day) plus green tea extract, methy-sulfonyl-methane (MSM) and vitamins and minerals (including vitamins A, B, C, D, E, folic acid and selenium) or placebo for 60 days [85]. Patients who received *Bacillus coagulans* experienced borderline statistically significant improvement from baseline in the Patient Pain Assessment score (*p* = 0.052) and statistically significant improvement from baseline in the Pain Scale (*p* = 0.046) compared to the placebo group.

### 5.2. Prebiotics

The concept of a *prebiotic* was first established in 1995 [86]. Later redefined as a selectively fermented ingredient that allows specific changes, both in the composition and/or activity of the GIT microflora that confers benefits upon host well-being and health [87,88]. Prebiotic classification of a compound requires it to meet a number of criteria, namely that it is non-digestible, fermented by intestinal microbiota and it selectively stimulates the growth and activity of intestinal bacteria, as demonstrated by *in vitro* and *in vivo* examination [89,90]. Prebiotics provide an energy source for growth of commensal bacteria already established in the GIT, thereby potentiating the beneficial actions of the GIT microbiome. This includes the synthesis of SCFAs that affect cell proliferation and differentiation, induce entero-endocrine peptide hormones (e.g., glucagon-like peptide 2), modulate inflammation (e.g., decrease TNF-α; increase IL-10) and influence leukocyte chemotaxis [91,92]. The extensive metabolic capacity of the gut microbiome however, enables it to ferment and metabolize substances beyond the typical non-digestible fibers, such as unabsorbed or undigested proteins, to produce biologically active compounds as either signaling- or nutrient compounds and precursors for Short Chain Fatty Acids (SCFAs) synthesis [92].

There are other classes of non-digestible dietary products that are not typically considered to be prebiotics. These include phenolic compounds from plants and known to be metabolized by GIT bacteria, producing various bioactive metabolites [93]. Early *in vitro* studies confirm that commensal bacterial species within the gut do ferment and metabolize glucosamine [94,95,96,97,98,99]. A large number of GIT microbiota genera and species are known to ferment glucosamine, including several *Lactobacilli* strains (*i.e.*, *L. casei*, *L. plantarum*, *L. acidophilus*, and *L. leichmanii*), *Streptococcus*, *Staphylococcus*, *Leuconostoc* [96], *Escherichia coli*, *Enterococcus faecalis*, *Proteus vulgaris* and *Bacillus coli* (from which *E. coli* is a descendant) [97,98]. Bacteria utilize amino sugars by acquiring them or synthesizing them to form peptidoglycans and lipopolysaccharides in the bacterial cell wall. GIT mucosal cells utilize glucosamine to synthesize and secrete the protective mucins along the whole length of the GIT tract [100]. The biosynthetic and degradative steps for glucosamine in bacteria are subjected to tight regulation [101] that leads to sufficient glucosamine biosynthesis for bacterial cell growth and survival [95,102,103,104]. It is suggested that any benefit demonstrated by glucosamine for osteoarthritic joints may be secondary to its effects on non-articular tissues, such as GIT barrier function and bacterial growth [105]. This potentially qualifies it as a prebiotic substance. Similarly, in limited studies examining the effect of green-lipped mussel GLM on GIT bacteria, suggest that GLM influence GIT bacterial profiles [13] and the anti-inflammatory effect exhibited by the GLM may involve its modulation first by gut commensal bacteria prior to inducing a local anti-inflammatory effect that spreads extra-intestinally to the joints. 

**Table 1:** Clinical trials with probiotics for the treatment of autoimmune arthritic diseases.

Study Participant Type (n = number of participants)	Probiotic strains employed and dose	Results	References
DBRCT RA (n = 21)	*Lactobacillus rhamnosus* GG (LGG) or placebo for 12 months. Dose: 5 × 10^9^ CFU/capsule/day/52 weeks	Although there were no statistical significant differences in the activity of RA, more subjects in the LGG group reported subjective well-being.	[82]
DBRCT Spondyloarthritis (n = 63)	*Streptoccocus salivarius* (1 × 10^8^ CFU/g), *Bifidobacterium lactis* (4 × 10^8^ CFU/g), *Lactobacillus acidophilus* (1 × 10^8^ CFU/g) Dose: 0.8 g (1 level tea spoonful) b.i.d./12 weeks	Probiotic therapy did not improve disease activity, function or quality of life. The probiotic group did however demonstrate an improvement in GIT symptoms within the 12-week period.	[83]
DBRCT RA (n = 29)	*Lactobacillus rhamnosus* GR-1 *Lactobacillus reuteri* RC-14 Dose: 2 × 10^9^ CFU/capsule/b.i.d./12 weeks	Although probiotics did not clinically improve RA as measured by the ACR20 there was functional improvement seen within the probiotic group compared to placebo.	[84]
DBRCT RA (n = 45)	*Bacillus coagulans* GBI-30, 6086 Dose: 2 × 10^9^ CFU/capsule/b.i.d./8 weeks	Results of this pilot study suggest that adjunctive treatment with *Bacillus coagulans* GBI-30, 6086 LAB probiotic appeared to be a safe and effective for patients diagnosed with RA.	[85]

DBRCT: Double Blind Randomized Controlled Trial; ACR20: American College of Rheumatology core set of disease activity measures for RA.

## 6. Mechanism of Action of Probiotics

Over the last six decades, oxidative stress has been proposed to play a major role in the development of chronic diseases such as inflammatory bowel diseases, cardiovascular diseases advocating that antioxidant strategies should become part of the treatment strategy [106]. We assert that this is incorrect. 

### 6.1. The Intracellular Second Messenger Role of ROS

**Figure 1:**
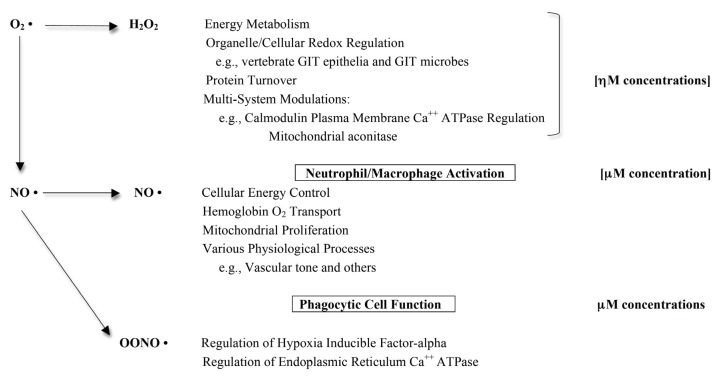
Synopsis of physiologically normal ROS/RNS regulatory functions for intracellular and extracellular interactions (adapted with permission from [107]).

Reactive oxygen species (ROS) are known to play a major role in maintaining normal physiological function [107] (Figure 1). The investigations on protein albumin thiol oxidations and serum protein carbonyl formations overestimate the damage that is attributed to ROS activity. 

This inference further nurtures support for the administration of antioxidant therapies. However, there are no reported clinical trials that support this conclusion. Our group [107] have previously considered and challenged the commonly-held view that proteins are randomly oxidized in an uncontrolled process by superoxide anion, hydrogen peroxide, nitric oxide and peroxynitrite, thereby contributing directly to the development of chronic diseases and the aging process. We concluded that this concept is not tenable and it is in error, misrepresenting stringently-regulated cellular redox metabolism.

The oxidation of protein amino acid residues, since their discovery some decades ago, has been almost universally reported as leading to protein inactivation and requiring mandatory proteolysis to prevent their deleterious cellular accumulation. It is clear that oxidatively-modified proteins do not simply arise as the result of random oxidative damage (hydroxylations of various amino acid residues, sulfoxidation of methionines, nitrosylations of sulphydryl groups and so on). There is an increasing number of situations where free radical protein modifications can be shown to be part of normal cellular regulatory signaling activity. As for example (i) the oxidation of only two of the seven specific methionine residues of calmodulin that is involved in the process of down-regulating plasma membrane Ca^++^ATPase and (ii) the turnover of the hypoxia-inducible factor-alpha (HIF**a**) and its proteasome degradation that is undoubtedly regulated by hydroxylation of its prolyl residues [107]. This is an ordered process involving signaling by the free radical system encompassed of superoxide anion, nitric oxide and peroxynitrite. 

We have reported [107] (as have others) that ROS generated by Nox enzymes have been shown to function as critical second messengers in multiple signal transduction pathways via the rapid and transient oxidative inactivation of a distinct class of sensor proteins bearing oxidant-sensitive thiol groups. 

Probiotic bacteria have been reported to temper a range of GIT physiological functions that include a regulated control over immune responses, epithelial barrier function and cellular proliferation [108]. The downstream mechanism that has been advanced for the GIT control of pathogens is (i) a direct anti-microbial action through assembly of bacteriocins or inhibitors of pathogen gene expression; (ii) competitive exclusion of pathogens by competing for binding sites or stimulation of epithelial barrier function; (iii) stimulation of immune responses via increases of sIgA and anti-inflammatory cytokines and the rescue and regulation of pro-inflammatory cytokines; and (iv) inhibition of virulence gene or protein expression in gastrointestinal pathogens [108]. The upstream mechanism that induces this complex control of pathogenic activity implicates ROS.

A recent study has demonstrated that some genera of human GIT bacteria can induce a rapid increase of ROS, eliciting a physiological response through the activation of epithelial NADPH oxidase-1 (Nox1) [109,110]. In addition, reports site *in vitro* experiments with epithelial cells that, when co-cultured with specific probiotic bacteria, show an increased and rapid oxidation reaction of soluble redox sinks, namely glutathione and thioredoxin [109,110]. This action indicates the presence of a regulated process. This effect was demonstrated as an increase in the oxido-reductase reaction of transcriptional factor activations such as nuclear factor kappa B (NFκB), NrF2 and the antioxidant response element, reflecting a cellular response to increased ROS production that is regulated [109,110]. This effect must be decisive in order to elicit a restrained anti-infective response with a minimal chance of pro-inflammatory damage to the tissue. These reactions define potent regulatory effects on host physiological functions that include immune function and intracellular signaling. 

The reported mechanisms of action of probiotics are similarly aligned acting to enhance the epithelial barrier, increased bacterial adhesion to the intestinal mucosa, with an attendant inhibition of pathogen adhesion to the competitive exclusion of pathogenic microorganisms [109,110,111,112,113,114,115]. Furthermore, probiotic strains have also been reported to generate a range of anti-microbial substances and to positively affect and modulate immune system function. Lee [112] has reported that the enteric commensal bacteria, by rapidly generating ROS, negotiate an acceptance by the GIT epithelia. Different strains of commensal bacteria can elicit markedly different levels of ROS from contacted cells. *Lactobacilli* are especially potent inducers of ROS generation in cultured cells and *in vivo*, though all bacteria tested have some ability to alter the intracellular oxido-reductase environment [111]. Yan [113] has reported that there are soluble factors that are produced by strains of *lactobacilli* that are capable of mediating beneficial effects *in vivo* inflammatory models. This result expands our understanding that there are ROS-stimulating bacteria that possess effective specific membrane components and or secreted factors that activate cellular ROS production to maintain homeostasis. 

### 6.2. ROS and the GIT Microbiome

It has been reported that redox signaling by microbial ROS formation is in response to microbial signals via formyl peptide receptors and the gut epithelial NADPH oxidase 1 (Nox1) [110]. As we have previously documented [107] ROS generated by Nox enzymes have been shown to function as essential second messengers in multiple signal transduction metabolic pathways through the rapid and transient oxidative inactivation of a distinct class of sensor proteins bearing oxidant-sensitive thiol groups. These redox sensitive proteins include tyrosine phosphatases that attend as regulators of the MAP kinase pathways, focal adhesion kinase [107,110]. These reports focuses our understanding on the importance of second messenger functionality for the maintenance of homeostasis and brings into serious question of the annulment of ROS by antioxidant supplements for the amelioration of chronic diseases such as CKD. The importance of recent investigations regarding probiotic/microbial-elicited ROS teaches that stimulated cellular proliferation and motility is strictly controlled and is a regulated signaling process for proper innate immunity and GIT barrier functionality [111,114,115]. The observations that the vertebrate epithelia of the intestinal tract supports a tolerable low-level inflammatory response toward the GIT microflora can be viewed as an adaptive activity that maintains homeostasis [116].

## 7. Conclusions

The regulatory role of the GIT microbiota in immune and inflammatory activity and the metabolic potential that it harbors provides a novel avenue of research for musculoskeletal diseases with potentially novel treatment options. 

Gut dysbiosis reflecting an imbalanced GIT microbiome that may be associated with impaired GIT mucosal barrier functions are associated with numerous adverse health concerns such as translocation of bacteria-derived lipopolysaccharides, accumulation of endotoxins and hyper-activation of the immune system. The host-microbiota through a series of complex cooperative tasks seeks to maintain homeostasis throughout.

Results from our clinical studies, has shown that in patients diagnosed with OA, the participants displayed GIT dysbiosis that was partially rescued by compounds such as glucosamine and green-lipped mussel extract [12,13]. The therapeutic efficacy of these compounds may be associated with the re-regulation of a dysbiotic gut that can subsequently influence immune and inflammatory activity. An adaptive tolerable activity by the commensal microbial cohort that enhances the GIT milieu in favor of the host is promoted. The microbial cohort in the GIT tolerates the host provided the milieu concurrently supports the microbial cohort organ. As such it would seem plausible that a mutually symbiotic relationship is in effect that maintains local and extra-intestinal inflammatory responses regulated. This co-operative microbial cohort-host activity may also provide beneficial effects in patients diagnosed with RA [52].

In this review commentary, we have also advanced support for the role of reactive oxygen species as the upstream mediators of the signaling message that commensal and probiotic bacteria elaborate, to maintain homeostasis whilst tolerating a symbiotic relationship with the host. The implication of the GIT microbiota in the pathogenesis of OA and RA and interventions with probiotics is in its infancy and presents a complex and intellectually challenging exercise.

## References

[1] Chong V., Wang C. (2008). Higher prevalence of gastrointestinal symptoms among patients with rheumatic disorders. Singapore Med. J..

[2] Wolfe F., Kong S., Watson D. (2000). Gastrointestinal symptoms and health related quality of life in patients with arthritis. J. Rheumatol..

[3] Zhang W., Moskowitz R., Nuki G., Abramson S., Altman R., Arden N., Bierma-Zeinstra S., Brandt K.D., Croft P., Doherty M. (2008). OARSI recommendations for the management of hip and knee osteoarthritis, Part II: OARSI evidence-based, expert consensus guidelines. Osteoarthr. Cartil..

[4] Jones R. (2001). Nonsteroidal anti-inflammatory drug prescribing: Past, present and future. Am. J. Med..

[5] Garcia Rodriguez L.A., Hernandez-Diaz S. (2001). Relative risk of upper gastrointestinal complications among users of acetaminophen and nonsteroidal anti-inflammatory drugs. Epidemiology.

[6] Rahme E., Pettitt D., LeLorier J. (2002). Determinants and sequelae associated with utilization of acetaminophen *versus* traditional nonsteroidal antiinflammatory drugs in an elderly population. Arthritis Rheum..

[7] Rahme E., Barkun A., Nedjar H., Gaugris S., Watson D. (2008). Hospitalization for upper and lower GI events associated with traditional NSAIDs and acetaminophen among the elderly in Quebec, Canada. Am. J. Gastroenterol..

[8] Scarpignato C., Hunt R. (2010). Nonsteroidal antiinflammatory drug-related injury to the gastrointestinal tract: Clinical picture, pathogenesis, and prevention. Gastroenterol. Clin. North Am..

[9] Upreti R., Kannan A., Pant A. (2010). Experimental impact of aspirin exposure on rat intestinal bacteria, epithelial cells and cell line. Hum. Exp. Toxicol..

[10] Cuzzolin L., Conforti A., Donini M., Adami A., del Soldato P., Benoni G. (1994). Effects on intestinal microflora, gastrointestinal tolerability and antiinflammatory efficacy of diclofenac and nitrofenac in adjuvant arthritic rats. Pharmacol. Res..

[11] Al-Janabi A.A. (2010). *In vitro* antibacterial activity of Ibuprofen and acetaminophen. J. Glob. Infect. Dis..

[12] Coulson S., Vecchio P., Gramotnev H., Vitetta L. (2012). Green-lipped mussel (*Perna canaliculus*) extract efficacy in knee osteoarthritis and improvement in gastrointestinal dysfunction: A pilot study. Inflammopharmacology.

[13] Coulson S., Butt H., Vecchio P., Gramotnev H., Vitetta L. (2013). Green-lipped mussel extract (*Perna canaliculus*) and glucosamine sulphate in patients with knee osteoarthritis: Therapeutic efficacy and effects on gastrointestinal microbiota profiles. Inflammopharmacology.

[14] Blaut M., Clavel T. (2007). Metabolic diversity of the intestinal microbiota: Implications for health and disease. J. Nutr..

[15] O’Hara A.M., Shanahan F. (2006). The gut flora as a forgotten organ. EMBO Rep..

[16] Bocci V. (1992). The neglected organ: Bacterial flora has a crucial immunostimulatory role. Perspect. Biol. Med..

[17] Goldin B. (1990). Intestinal microflora: Metabolism of drugs and carcinogens. Ann. Med..

[18] Laparra J.M., Sanz Y. (2010). Interactions of gut microbiota with functional food components and nutraceuticals. Pharmacol. Res..

[19] Gul’neva M., Noskov S.M. (2011). Colonic microbial biocenosis in rheumatoid arthritis. Klin. Med. (Mosk).

[20] Qin J., Li R., Raes J., Arumugam M., Burgdorf K.S., Manichanh C., Nielsen T., Pons N., Levenez F., Yamada T. (2010). A human gut microbial gene catalogue established by metagenomic sequencing. Nature.

[21] Ley R., Peterson D., Gordon J. (2006). Ecological and evolutionary forces shaping microbial diversity in the human intestine. Cell.

[22] Egert M., de Graaf A.A., Smidt H., de Vos W.M., Venema K. (2006). Beyond diversity: Functional microbiomics of the human colon. Trends Microbiol..

[23] Nicholson J.K., Holmes E., Kinross J., Burcelin R., Gibson G., Jia W., Pettersson S. (2012). Host-gut microbiota metabolic interactions. Science.

[24] Börnigen D., Morgan X.C., Franzosa E.A., Ren B., Xavier R.J., Garrett W.S., Huttenhower C. (2013). Functional profiling of the gut microbiome in disease-associated inflammation. Genome Med..

[25] Tremaroli V., Bäckhed F. (2012). Functional interactions between the gut microbiota and host metabolism. Nature.

[26] Candela M., Biagi E., Maccaferri S., Turroni S., Brigidi P. (2012). Intestinal microbiota is a plastic factor responding to environmental changes. Trends Microbiol..

[27] Wu G.D., Chen J., Hoffmann C., Bittinger K., Chen Y.Y., Keilbaugh S.A., Bewtra M., Knights D., Walters W.A., Knight R. (2011). Linking long-term dietary patterns with gut microbial enterotypes. Science.

[28] Arumugam M., Raes J., Pelletier E., Le Paslier D., Yamada T., Mende D.R., Fernandes G.R., Tap J., Bruls T., Batto J.M. (2011). Enterotypes of the human gut microbiome. Nature.

[29] Umesaki Y., Setoyama H., Matsumoto S., Imaoka A., Itoh K. (1999). Differential roles of segmented filamentous bacteria and clostridia in development of the intestinal immune system. Infect. Immun..

[30] Kelly D., Conway S., Aminov R. (2005). Commensal gut bacteria: Mechanisms of immune modulation. Trends Immunol..

[31] Fasano A. (2002). Toxins and the gut: Role in human disease. Gut.

[32] Bengmark S. (2013). Gut microbiota, immune development and function. Pharmacol. Res..

[33] Stecher B., Maier L., Hardt W.D. (2013). “Blooming” in the gut: How dysbiosis might contribute to pathogen evolution. Nat. Rev. Microbiol..

[34] Owen J.L., Mohamadzadeh M. (2013). Microbial activation of gut dendritic cells and the control of mucosal immunity. J. Interferon Cytokine Res..

[35] Cani P.D., Delzenne N.M. (2007). Gut microflora as a target for energy and metabolic homeostasis. Curr. Opin. Clin. Nutr. Metab. Care.

[36] Mackay I.R., Rosen F.S., Zinkernagel R.M. (2001). Maternal antibodies, childhood infections, and autoimmune diseases. N. Engl. J. Med..

[37] Sironi M., Clerici M. (2010). The hygiene hypothesis: An evolutionary perspective. Microbes Infect..

[38] Bach J.F. (2002). The effect of infections on susceptibility to autoimmune and allergic diseases. N. Engl. J. Med..

[39] Shanahan F. (2005). Physiological basis for novel drug therapies used to treat the inflammatory bowel disease. I. Pathophysiological basis and prospects for probiotic therapy in inflammatory bowel disease. Am. J. Physiol. Gastrointestinal. Liver Physiol..

[40] Rescigno M., Urbano M., Valzasina B., Francolini M., Rotta G., Bonasio R., Granucci F., Kraehenbuhl J.P., Ricciardi-Castagnoli P. (2001). Dendritic cells express tight junction proteins and penetrate gut epithelial monolayers to sample bacteria. Nat. Immunol..

[41] Lambrecht B., Salomon B., Klatzmann D., Pauwels R. (1998). Dendritic cells are required for the development of chronic eosinophilic airway inflammation in response to inhaled antigen in sensitized mice. J. Immunol..

[42] Banchereau J., Briere F., Caux C., Davoust J., Lebecque S., Liu Y., Pulendran B., Palucka K. (2000). Immunology of dendritic cells. Annu. Rev. Immunol..

[43] Cario E. (2005). Bacterial interactions with cells of the intestinal mucosa: Toll-like receptors and NOD2. Gut.

[44] Rozee K., Cooper D., Lam K., Costerton J. (1982). Microbial flora of the mouse ileum mucous layer and epithelial surface. Appl. Environ. Microbiol..

[45] Sanford S. (1991). Light and electron microscopic observations of a segmented filamentous bacterium attached to the mucosa of the terminal ileum of pigs. J. Vet. Diagn. Invest..

[46] Atarashi K., Tanoue T., Shima T., Imaoka A., Kuwahara T., Momose Y., Cheng G., Yamasaki S., Saitom T., Ohba Y. (2011). Induction of colonic regulatory T cells by indigenous Clostridium species. Science.

[47] O’Hara A.M., O’Regan P., Fanning A., O’Mahony C., MacSharry J., Lyons A., Bienenstock J., O’Mahony L., Shanahan F. (2006). Functional modulation of human intestinal epithelial cells responses by *Bifidobacterium infantis* and *Lactobacillus salivarius*. Immunology.

[48] Wills-Karp M., Santeliz J., Karp C.L. (2001). The germless theory of allergic disease: Revisiting the hygiene hypothesis. Nat. Rev. Immunol..

[49] O’Neill L.A. (2006). How Toll-like receptors signal: What we know and what we don’t know. Curr. Opin. Immunol..

[50] Pasare C., Medzhitov R. (2004). Toll-dependent control mechanisms of CD4 T cell activation. Immunity.

[51] Hussaarts L., van der Vlugt L.E., Yazdanbakhsh M., Smits H.H. (2011). Regulatory B-cell induction by helminths: Implications for allergic disease. J. Allergy Clin. Immunol..

[52] Scher J.U., Abramson S.B. (2011). The microbiome and rheumatoid arthritis. Nat. Rev. Rheumatol..

[53] Vaahtovuo J., Munukka E., Korkeamäki M., Luukkainen R., Toivanen P. (2008). Fecal microbiota in early rheumatoid arthritis. J. Rheumatol..

[54] Toivanen P. (2003). Normal intestinal microbiota in the aetiopathogenesis of rheumatoid arthritis. Ann. Rheum. Dis..

[55] Wu H.J., Ivanov I.I., Darce J., Hattori K., Shima T., Umesaki Y., Littman D.R., Benoist C., Mathis D. (2010). Gut-residing segmented filamentous bacteria drive autoimmune arthritis via T helper 17 cells. Immunity.

[56] Jarchum I., Pamer E.G. (2011). Regulation of innate and adaptive immunity by the commensal microbiota. Curr. Opin. Immunol..

[57] Stepankova R., Powrie F., Kofronova O., Kozakova H., Hudcovic T., Hrncir T., Uhlig H., Read S., Rehakova Z., Benada O. (2007). Segmented filamentous bacteria in a defined bacterial cocktail induce intestinal inflammation in SCID mice reconstituted with CD45RBhigh CD4+ T cells. Inflamm. Bowel Dis..

[58] Han Y.W., Wang X. (2013). Mobile microbiome: Oral bacteria in extra-oral infections and inflammation. J. Dent. Res..

[59] Haffner R.S., Keyßer G., Sch€afer C., Stein J.M., Schaller H.G., Wienke A., Strauss H., Heide S., Schulz S. (2013). Detection of oral bacterial DNA in synovial fluid. J. Clin. Periodontol..

[60] Yeoh N., Burton J.P., Suppiah P., Reid G., Stebbings S. (2013). The role of the microbiome in rheumatic conditions. Curr. Rheumatol. Rep..

[61] Myneni S.R., Settem R.P., Sharma A. (2013). Bacteria take control of tolls and T cells to destruct jaw bone. Immunol. Invest..

[62] Martinez-Martinez R.E., Abud-Mendoza C., Patino-Marin N., Rizo-Rodriguez J.C., Little J.W., Loyola-Rodriguez J.P. (2009). Detection of periodontal bacterial DNA in serum and synovial fluid in refractory rheumatoid arthritis patients. J. Clin. Periodontol..

[63] Moen K., Brun J.G., Madland T.M., Tynning T., Jonsson R. (2003). Immunoglobulin G and A antibody responses to *Bacteroides forsythus* and *Prevotella intermedia* in sera and synovial fluids of arthritis patients. Clin. Diagn. Lab. Immunol..

[64] Kempsell K., Cox C., Hurle M., Wong A., Wilkie S., Zanders E., Gaston J.S., Crowe J.S. (2000). Reverse transcriptase-PCR analysis of bacterial rRNA for detection and characterization of bacterial species in arthritis synovial tissue. Infect. Immun..

[65] Gerard H.C., Wang Z., Wang G.F., El-Gabalawy H., Goldbach-Mansky R., Li Y., Majeed W., Zhang H., Ngai N., Hudson A.P. (2001). Chromosomal DNA from a variety of bacterial species is present in synovial tissue from patients with various forms of arthritis. Arthritis Rheum..

[66] Van der Heijden I.M., Wilbrink B., Tchetverikov I., Schrijver I.A., Schouls L.M., Hazenberg M.P., Breedveld F.C., Tak P.P. (2000). Presence of bacterial DNA and bacterial peptidoglycans in joints of patients with rheumatoid arthritis and other arthritides. Arthritis Rheum..

[67] Siala M., Gdoura R., Fourati H., Rihl M., Jaulhac B., Younes M., Sibilia J., Baklouti S., Bargaoui N., Sellami S. (2009). Broad-range PCR, cloning and sequencing of the full 16S rRNA gene for detection of bacterial DNA in synovial fluid samples of Tunisian patients with reactive and undifferentiated arthritis. Arthritis Res. Ther..

[68] Olmez N., Wang G.F., Li Y., Zhang H., Schumacher H.R. (2001). Chlamydial nucleic acids in synovium in osteoarthritis: What are the implications?. J. Rheumatol..

[69] Carter J.D., Gerard H.C., Espinoza L.R., Ricca L.R., Valeriano J., Snelgrove J., Oszust C., Vasey F.B., Hudson A.P. (2009). Chlamydiae as etiologic agents in chronic undifferentiated spondylarthritis. Arthritis Rheum..

[70] Gerard H.C., Stanich J.A., Whittum-Hudson J.A., Schumacher H.R., Carter J.D., Hudson A.P. (2010). Patients with Chlamydia-associated arthritis have ocular (trachoma), not genital, serovars of *C. trachomatis* in synovial tissue. Microb. Pathog..

[71] Levy O., Iyer S., Atoun E., Peter N., Hous N., Cash D., Musa F., Narvani A.A. (2013). Propionibacterium acnes: An underestimated etiology in the pathogenesis of osteoarthritis?. J. Shoulder Elbow Surg..

[72] Schaeverbeke T., Lequen L., de Barbeyrac B., Labbe L., Bebear C.M., Morrier Y., Bannwarth B., Bébéar C., Dehais J. (1998). Propionibacterium acnes isolated from synovial tissue and fluid in a patient with oligoarthritis associated with acne and pustulosis. Arthritis Rheum..

[73] Trimble B.S., Evers C.J., Ballaron S.A., Young J.M. (1987). Intraarticular injection of Propionibacterium acnes causes an erosive arthritis in rats. Agents Actions.

[74] Sartor R.B. (2005). Mechanisms of disease: Pathogenesis of Crohn’s disease and ulcerative colitis. Nat. Clin. Pract. Gastroenterol. Hepatol..

[75] Fuller R. (1989). Probiotics in man and animals. J. Appl. Bacteriol..

[76] Vitetta L., Briskey D., Hayes E., Shing C., Peake J. (2013). A review of the pharmacobiotic regulation of gastrointestinal inflammation by probiotics, commensal bacteria and prebiotics. Inflammopharmacology.

[77] Reid G., Jass J., Sebulsky M., McCromick J. (2003). Potential uses of probiotics in clinical practice. Clin. Microbiol. Rev..

[78] So J.S., Kwon H.K., Lee C.G., Yi H.J., Park J.A., Lim S.Y., Hwang K.C., Jeon Y.H., Im S.H. (2008). *Lactobacillus casei* suppresses experimental arthritis by down-regulating T helper 1 effector functions. Mol. Immunol..

[79] So J.S., Lee C.G., Kwon H.K., Yi H.J., Chae C.S., Park J.A., Hwang K.C., Im S.H. (2008). *Lactobacillus casei* potentiates induction of oral tolerance in experimental arthritis. Mol. Immunol..

[80] Amdekar S., Singh V., Singh R., Sharma P., Keshav P., Kumar A. (2011). *Lactobacillus casei* reduces the inflammatory joint damage associated with collagen-induced arthritis (CIA) by reducing the pro-inflammatory cytokines: *Lactobacillus casei*: COX-2 inhibitor. J. Clin. Immunol..

[81] So J.S., Song M.K., Kwon H.K., Lee C.G., Chae C.S., Sahoo A., Jash A., Lee S.H., Park Z.Y., Im S.H. (2011). *Lactobacillus casei* enhances type II collagen/glucosamine-mediated suppression of inflammatory responses in experimental osteoarthritis. Life Sci..

[82] Hatakka K., Martio J., Korpela M., Herranen M., Poussa T., Laasanen T., Saxelin M., Vapaatalo H., Moilanen E., Korpela R. (2003). Effects of probiotc therapy on activity and activation of mild rheumatoid arthritis—A pilot study. Scand. J. Rheumatol..

[83] Jenks K., Stebbings S., Burton J., Schultz M., Herbison P., Highton J. (2010). Probiotic therapy for the treatment of Spondyloarthritis: A randomized controlled study. J. Rheumatol..

[84] Pineda Mde L., Thompson S.F., Summers K., de Leon F., Pope J., Reid G. (2011). A randomized, double-blinded, placebo-controlled pilot study of probiotics in active rheumatoid arthritis. Med. Sci. Monit..

[85] Mandel D.R., Eichas K., Holmes J. (2010). *Bacillus coagulans*: A viable adjunct therapy for relieving symptoms of rheumatoid arthritis according to a randomized, controlled trial. BMC Complement. Altern. Med..

[86] Gibson G.R., Roberfroid M.B. (1995). Dietary modulation of the colonic microbiota: Introducing the concept of prebiotics. J. Nutr..

[87] Gibson G.R., Probert H.M., van Loo J.A.E., Roberfroid M.B. (2004). Dietary modulation of the human colonic microbiota: Updating the concept of prebiotics. Nutr. Res. Rev..

[88] Roberfroid M. (2007). Prebiotics: The concept revisited. J. Nutr..

[89] De Vrese M., Schrezenmeir J. (2008). Probiotics, prebiotics, and synbiotics. Adv. Biochem. Eng. Biotechnol..

[90] Vitetta L., Sali A. (2008). Probiotics, prebiotics and gastrointestinal health. Med. Today.

[91] Everard A., Cani P. (2013). Diabetes, obesity and gut microbiota. Best Pract. Res. Clin. Gastroenterol..

[92] Soldavini J., Kaunitz J.D. (2013). Pathobiology and potential therapeutic value of intestinal short-chain fatty acids in gut inflammation and obesity. Dig. Dis. Sci..

[93] Geurts L., Neyrinck A.M., Delzenne N.M., Knauf C., Cani P.D. (2013). Gut microbiota controls adipose tissue expansion, gut barrier and glucose metabolism: Novel insights into molecular targets and interventions using prebiotics. Benef. Microbes.

[94] Aghazadeh-Habashi A., Sattari S., Pasutto F.M., Jamali F. (2002). Single dose pharmacokinetics and bioavailability of glucosamine in the rat. J. Pharm. Pharmaceut. Sci..

[95] Foley S., Stolarczyk E., Mouni F., Brassart C., Vidal O., Aissi E., Bouquelet S., Krzewinski F. (2008). Characterisation of glutamine fructose-6-phosphate amidotransferase (EC 2.6.1.16) and *N*-acetylglucosamine metabolism in Bifidobacterium. Arch. Microbiol..

[96] Koser S., Tribby I., Stuedell J. (1961). Glucosamine utilization by some lactic acid bacteria. J. Infect. Dis..

[97] Wolfe J., Nakada H. (1956). Glucosamine degradation by *Escherichia coli*. II. The isomeric conversion of glucosamine 6-PO4 to fructose 6-PO4 and ammonia. Arch. Biochem. Biophys..

[98] Lutwak-Mann C. (1941). Enzymatic decomposition of amino-sugars. Biochem. J..

[99] Faulkner P., Quastel J. (1956). Anaerobic deamination of D-glucosamine by bacterial and brain extracts. Nature.

[100] Whitehouse M., Butters D. (1999). Non-NSAID overt-the-counter (OTC) remedies for arthritis: Good, bad or indifferent?. Inflammopharmacology.

[101] Alvarez-Anorve L., Calcagno M., Plumbridge J. (2005). Why does *Escherichia coli* grow more slowly on glucosamine than on *N*-acetylglucosamine? Effects of enzyme levels and allosteric activation of GlcN6P deaminase (NagB) on growth rates. J. Bacteriol..

[102] Plumbridge J., Cochet O., Souza J., Altamirano M., Calcagno M., Badet B. (1993). Coordinates regulation of amino sugar-synthesizing and degrading ezymes in *Eschericia coli* K-12. J. Bacteriol..

[103] Plumbridge J. (1995). Co-ordinated regulation of amino sugar biosynthesis and degradation: The NagC repressor acts as both an activator and a repressor for the transcription of the glmUS operon and requires two separated NagC binding sites. EMBO J..

[104] Vogler A., Trentmann S., Lengeler J. (1989). Alternative route for biosynthesis of amino sugars in *Eschericia coli* K-12 mutants of a catabolic isomerase. J. Bacteriol..

[105] Laverty S., Sandy J., Celeste C., Vachon P., Marier J., Plaas A. (2005). Synovial fluid levels and serum pharmacokinetics in a large animal model following treatment with oral glucosamine at clinically relevant doses. Arthritis Rheum..

[106] Kim Y.W., West X.Z., Byzova T.V. (2013). Inflammation and oxidative stress in angiogenesis and vascular disease. J. Mol. Med. (Berl.).

[107] Linnane A.W., Kios M., Vitetta L. (2007). Healthy aging: Regulation of the metabolome by cellular redox modulation and prooxidant signaling systems: The essential roles of superoxide anion and hydrogen peroxide. Biogerontology.

[108] Bermudez-Brito M., Plaza-Diaz J., Munoz-Quezada S., Gomez-Llorente C., Gil A. (2012). Probiotic mechanisms of action. Ann. Nutr. Metab..

[109] Amalaradjou M.A., Bhunia A.K. (2012). Modern approaches in probiotics research to control foodborne pathogens. Adv. Food Nutr. Res..

[110] Neish A.S. (2013). Redox signaling mediated by the gut microbiota. Free Radic. Res..

[111] Lin P.W., Myers L.E., Ray L., Song S.C., Nasr T.R., Berardinelli A.J., Kundu K., Murthy N., Hansen J.M., Neish A.S. (2009). *Lactobacillus rhamnosus* blocks inflammatory signaling *in vivo* via reactive oxygen species generation. Free Radic. Biol. Med..

[112] Lee W.J. (2008). Bacterial-modulated signaling pathways in gut homeostasis. Sci. Signal..

[113] Yan F., Cao H., Cover T.L., Whitehead R., Washington M.K., Polk D.B. (2007). Soluble proteins produced by probiotic bacteria regulate intestinal epithelial cell survival and growth. Gastroenterology.

[114] Patel R.M., Myers L.S., Kurundkar A.R., Maheshwari A., Nusrat A., Lin P.W. (2012). Probiotic bacteria induce maturation of intestinal claudin 3 expression and barrier function. Am. J. Pathol..

[115] Collier-Hyams L.S., Sloane V., Batten B.C., Neish A.S. (2005). Cutting edge: Bacterial modulation of epithelial signaling via changes in neddylation of cullin-1. J. Immunol..

[116] Neish A.S., Gewirtz A.T., Zeng H., Young A.N., Hobert M.E., Karmali V., Rao A.S., Madara J.L. (2000). Prokaryotic regulation of epithelial responses by inhibition of I kappa B-alpha ubiquitination. Science.

